# VTA GABA Neurons at the Interface of Stress and Reward

**DOI:** 10.3389/fncir.2019.00078

**Published:** 2019-12-05

**Authors:** Chloé Bouarab, Brittney Thompson, Abigail M. Polter

**Affiliations:** Department of Pharmacology and Physiology, Institute for Neuroscience, George Washington University School of Medicine and Health Sciences, Washington, DC, United States

**Keywords:** ventral tegmental area (VTA), GABA, reward, stress, circuits

## Abstract

The ventral tegmental area (VTA) is best known for its robust dopaminergic projections to forebrain regions and their critical role in regulating reward, motivation, cognition, and aversion. However, the VTA is not only made of dopamine (DA) cells, as approximately 30% of cells in the VTA are GABA neurons. These neurons play a dual role, as VTA GABA neurons provide both local inhibition of VTA DA neurons and long-range inhibition of several distal brain regions. VTA GABA neurons have increasingly been recognized as potent mediators of reward and aversion in their own right, as well as potential targets for the treatment of addiction, depression, and other stress-linked disorders. In this review article, we dissect the circuit architecture, physiology, and behavioral roles of VTA GABA neurons and suggest critical gaps to be addressed.

## Introduction

The ventral tegmental area (VTA) is a hub of the mesocorticolimbic circuitry that plays a significant role in reward, motivation, cognition, and aversion. Dopaminergic (DA) neurons, which make up 65% of neurons in the VTA, have been the primary focus of research into this brain region. We have long known that the VTA is a heterogeneous structure, with multiple cell types (Yim and Mogenson, 1980; Gysling and Wang, 1983; Grace and Onn, 1989; Johnson and North, 1992). Recent years have seen a surge of interest in the non-dopaminergic cells of the VTA, the majority of which are GABA neurons (Nair-Roberts et al., 2008). VTA GABA neurons have diverse functions, influencing dopaminergic activity through local inhibitory control as well as exerting dopamine (DA)-independent effects through projections to distal brain regions. Recent work has revealed complex roles for these neurons, identifying them as critical mediators of reward, aversion, and associative learning. VTA GABA neurons are also strongly modulated by drugs of abuse and stress, pointing towards potential roles in substance use and stress-related neuropsychiatric disorders. Despite recent advances, there are still lingering questions about VTA GABA neurons to be answered. In this review article, we outline what is known about the anatomy, physiology, and behavioral roles of VTA GABA neurons, and highlight some important next steps for the field.

## Circuit Architecture of VTA GABA Neurons

### Microcircuit Organization of VTA GABA Neurons

Relatively little is known about the local architecture of GABA neurons within the VTA, particularly when compared to our understanding of inhibitory networks within the forebrain regions.

GABA neurons are found throughout the VTA, however, they differ in abundance along medial-lateral and rostral-caudal gradients. In general GABA neurons are more abundant in the rostral and medial regions of the VTA while DA cells are found more in caudal and lateral regions (Ciccarelli et al., 2012; Morales and Margolis, 2017; Root et al., 2018). Ultrastructural and electrophysiological data has repeatedly shown that local VTA GABA neurons synapse onto VTA DA neurons and provide a significant source of inhibitory control (Omelchenko and Sesack, 2009; Matsui and Williams, 2011; Tan et al., 2012; van Zessen et al., 2012; Bocklisch et al., 2013; Matsui et al., 2014; Simmons et al., 2017; Polter et al., 2018). Ultrastructural data shows that synapses arising from VTA GABA neurons are primarily found on proximal dendrites of VTA DA neurons. These synapses are therefore likely to exert a level of inhibitory control over DA neurons that allows coordination of the activity of local cells but will have a considerably weaker effect than inhibitory inputs or excitatory inputs that synapse directly onto the cell body, such as the laterodorsal tegmentum and the ventral pallidum (Omelchenko and Sesack, 2005, 2009).

While there is clear evidence that VTA GABA neurons synapse onto and inhibit VTA DA neurons, the precise organization of this inhibition is still unclear. VTA DA neurons are highly heterogeneous. Discrete populations of DA neurons project to distinct areas and exhibit unique physiological characteristics, synaptic inputs and behavioral roles (Ford et al., 2006; Margolis et al., 2006; Lammel et al., 2008, 2012, 2014; Baimel and Borgland, 2015; Beier et al., 2015). Much remains unclear, however, about the relationship between different subclasses of DA neurons and their inhibitory neighbors. A recent study provided the first glimpse, suggesting that DA neurons projecting to the medial and lateral nucleus accumbens (NAc) receive quantitatively different local inhibition, with the lateral NAc projectors receiving considerably stronger spontaneous (and likely local) inhibitory input compared to medial NAc projectors (Yang et al., 2018). It is unknown if these differences arise from simply a difference in the number of synapses from the same source or if these subsets of DA neurons are contacted by distinct GABAergic neurons. Likewise, little is known about local connections between VTA GABA neurons and DA neurons projecting to the amygdala or prefrontal cortex (PFC). It seems clear that at minimum, local inhibition is not equally distributed among DA neurons with differing outputs. Furthermore, VTA GABA neurons make connections onto other VTA GABA neurons (Omelchenko and Sesack, 2009; Tan et al., 2012; Polter et al., 2018), but it is still unknown if these are reciprocal connections between neurons that also regulate DA neurons or two distinct subclasses of interneurons.

GABA neurons in the VTA have typically been identified by the presence of glutamic acid decarboxylase, a catalytic enzyme required for synthesis of GABA in most neurons. In mammals, GAD exists in two isoforms, GAD1 (GAD67) and GAD2 (GAD65; Erlander et al., 1991). VTA GABAergic neurons can also be identified by the presence of the vesicular GABA transporter (VGAT; Wojcik et al., 2006). Identifying sub-classes of VTA GABA neurons has been highly difficult, as canonical markers of interneuron subtypes from forebrain regions do not appear to map onto the VTA. Subsets of VTA GABA neurons have been found to contain corticotrophin-releasing factor-binding protein (Wang and Morales, 2008) or cholecystokinin (Olson and Nestler, 2007; Merrill et al., 2015), while others have been shown to respond specifically to DA receptors D2 or μ-opioid receptor activation (Margolis et al., 2012; for review, see Morales and Margolis, 2017). A study of VTA GABA neuron expression of calcium-binding proteins found that several of these proteins often co-express in a single neuron and that they are also expressed in DA neurons (Merrill et al., 2015; Table 1).

**Table 1:** Putative markers for subtypes of ventral tegmental area (VTA) GABA neurons.

Marker	Protein function	DA neurons	GABA neurons	Localization	Source
Parvalbumin	Calcium Binding Protein	+	+++	Rostromedial VTA	Olson and Nestler (2007) and Merrill et al. (2015)
Calbindin	Calcium Binding Protein	+	+++	PBP and midline nuclei	Olson and Nestler (2007) and Merrill et al. (2015)
Calretinin	Calcium Binding Protein	+	+++	Lateral PBP	Olson and Nestler (2007) and Merrill et al. (2015)
Cholecystokinin	peptide hormone	++	+++	PBP and C. Li	Olson and Nestler (2007) and Merrill et al. (2015)
Cerebellin 4 precursor	Secreted protein	+	++	PN and medial PBP	Paul et al. (2019)
Relaxin/insulin-like family peptide receptor 3	GPCR	+	+++	Rostromedial VTA	Paul et al. (2019)
RAR-related orphan receptor A	Nuclear hormone receptor	+	++	Sparsely distributed	Paul et al. (2019)
G-protein coupled receptor 101	GPCR	+	++	Sparsely distributed	Paul et al. (2019)
Neuropilin 2	Transmembrane receptor	N/A	N/A	Sparsely distributed in caudal VTA, primarily expressed in TH- processes	Paul et al. (2019)
Thyrotropin releasing hormone	Hormone	N/A	N/A	Sparsely distributed throughout VTA, Primarily expressed in TH-processes	Paul et al. (2019)
Neuronal nitric oxide synthase	Enzyme	-	++	PBP (GABAergic interneurons) and R. Li (glutamatergic projection neurons)	Paul et al. (2018)

*+, expressed in <25% of cell type ++, 25–50% of cell type +++, >50%+ of cell type. PBP, parabrachial pigmented area; PN, paranigral nucleus; C. Li, caudal linear nucleus; R. Li, rostral linear nucleus*.

Despite the failure of traditional markers to distinguish between VTA GABA neurons, the high degree of heterogeneity strongly suggests that VTA GABA neurons may have multiple subclasses. Several recent studies have attempted to define novel markers for subclasses of VTA GABA neurons. Developmental approaches have yielded insights into the heterogeneity of midbrain GABA neurons, suggesting that there are 3–4 subgroups of inhibitory neurons that populate the VTA, and an additional group that populates the rostromedial tegmental nucleus (RMTg; Lahti et al., 2016). However, these cells have many overlapping markers, which prevents gaining genetic access to a specific subtype. Recent work (Paul et al., 2018, 2019) identified seven promising candidates for labeling sub-groups of VTA GABA neurons (nNOS, CBLN4, GPR101, RORA, RXFP3, NRP2 and TRH; Table 1) by examining transcripts enriched in either GABAergic or DAergic neuronal populations in the VTA. While it remains to be seen how well these markers map onto functional classes of neurons, this is an important first step in defining subtypes of VTA GABA neurons.

One recent advance is the identification of the RMTg nucleus as a distinct brain region from the VTA (Jhou et al., 2009a). The RMTg, also referred to as the tail of the VTA, is located caudal to the VTA and dorsolateral to the caudal half of the interpeduncular nucleus (Jhou et al., 2009a; Kaufling et al., 2009; Smith et al., 2019). Due to its dense population of GABA neurons and its location, in which neurons from the RMTg intermingle with the caudal portion of the VTA, the RMTg was long considered to be an extension of the VTA. However, recent work shows that there are significant anatomical, genetic, and functional differences between VTA GABA neurons and RMTg neurons (Jhou et al., 2009a; Bourdy and Barrot, 2012; Lahti et al., 2016; Simmons et al., 2017; Polter et al., 2018; Smith et al., 2019). RMTg neurons, unlike VTA GABA neurons, strongly express fos protein after psychostimulant injection (Scammell et al., 2000; Perrotti et al., 2005; Geisler et al., 2008; Jhou et al., 2009b) and in response to aversive stimuli (Jhou et al., 2009a). They express neuronal markers such as somatostatin and the transcription factor FoxP1 (Lahti et al., 2016), and have differing basal synaptic properties and expression of synaptic plasticity (Simmons et al., 2017; Polter et al., 2018). In light of these data, it will be important to revisit older studies that do not distinguish between these two regions, and to develop genetic tools to clearly separate RMTg neurons from VTA GABA neurons. While this review does not include a thorough overview of studies of the RMTg (for review of the structure and function of the RMTg see Barrot et al., 2012; Bourdy and Barrot, 2012), many studies cited here do not distinguish between the RMTg and the VTA proper, therefore conclusions about VTA GABA neurons may, in fact, be due to a combination of VTA and RMTg GABA neurons.

### Afferents and Efferents of VTA GABA Neurons

**Figure 1:**
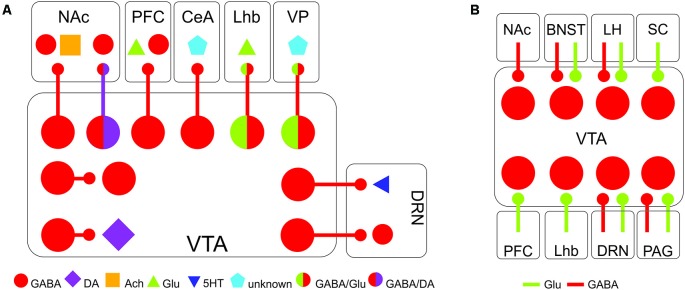
Major afferents and efferents of VTA GABA neurons. **(A)** Major projection targets of VTA GABA neurons within and beyond the VTA. **(B)** Major inputs onto VTA GABA neurons. Abbreviations: BNST, bed nucleus of the stria terminalis; CeA, central amygdala; DRN, dorsal raphe nucleus; LHb, lateral habenula; LH, lateral hypothalamus; NAc, nucleus accumbens; PAG, periaqueductal gray; PFC, prefrontal cortex; SC, superior colliculus; VP, ventral pallidum; VTA, ventral tegmental area.

GABA neurons of the VTA receive inhibitory, excitatory, and neuromodulatory inputs from throughout the brain (Morales and Margolis, 2017; Figure 1A). These inputs come from regions implicated in energy homeostasis, processing of rewards and threats, stress responses, and higher cognitive function, indicating that VTA GABA neurons are poised to integrate information about external occurrences and internal state to regulate behavior. Qualitatively, these inputs are largely similar to inputs received by DA neurons, although there are quantitative differences in the relative number of inputs GABA and DA neurons receive from the same region (Beier et al., 2015, 2019; Faget et al., 2016). Inputs to VTA GABA neurons show a higher degree of convergence than VTA DA neurons, with one presynaptic cell synapsing onto multiple VTA GABA neurons. This suggests that VTA GABA neurons can be uniformly controlled by afferent inputs, promoting synchronous activity (Faget et al., 2016). There is also evidence that brain regions can send projections to VTA DA and GABA neurons from distinct cells. For example, projections from the PFC to the VTA are composed of two distinct populations of neurons, one that targets VTA GABA neurons, and one that targets VTA DA neurons (Sesack and Pickel, 1992; Carr and Sesack, 2000).

VTA GABA neurons receive a number of inhibitory afferents, allowing the regulation of VTA DA neurons through disinhibition. Inhibitory inputs from the bed nucleus of the stria terminalis (BNST; Jennings et al., 2013), lateral hypothalamus (Nieh et al., 2016), medial preoptic area (MPOA, McHenry et al., 2017) and NAc (Bocklisch et al., 2013; Edwards et al., 2017; Yang et al., 2018) all primarily target inhibitory neurons within the VTA. By silencing local interneurons, these inputs increase the firing of DA neurons. In this configuration, the local VTA interneurons serve as a gate, controlling the flow of information through the VTA. By receiving inhibitory and excitatory input from such a wide array of inputs, VTA GABA neurons are likely to be a site of integration of diverse signals from throughout the brain.

In addition to local interneurons, a subset of VTA GABA neurons has efferents that project throughout the brain (Figure 1B). VTA GABA neurons project to the NAc, the ventral pallidum, the PFC, the central amygdala (CeA), the lateral habenula (LHb), and the dorsal raphe nucleus (DRN), among other regions (Van Bockstaele and Pickel, 1995; Brown et al., 2012; Creed et al., 2014; Taylor et al., 2014; Edwards et al., 2017; Morales and Margolis, 2017; Breton et al., 2019; Li et al., 2019). These projecting neurons likely exist in distinct sub-classes, much like neighboring dopaminergic neurons (Lammel et al., 2014), forming parallel circuits that regulate different facets of reward and aversion. Recent anatomical studies support this concept, as they have shown that GABA neurons projecting to different targets are spatially organized within the VTA, suggesting at least some segregation of neurons with different outputs (Breton et al., 2019).

Many of these projections have been identified anatomically, with little functional characterization of cellular properties of these neurons or synaptic properties of their terminals. This is particularly important as the firing rate, strength of synapses and the postsynaptic target contribute greatly to the ultimate physiological and behavioral output of the projection. A recent example of this is that of the VTA projection to the DRN, which contains two distinct GABAergic projections, one originating in the rostral VTA, which targets DRN GABA neurons and one in the caudal VTA, which targets DRN serotonergic neurons (Li et al., 2019). This highlights the enormous potential complexity of VTA GABA projection neurons, and the importance of functional studies to complement anatomical data.

## Physiological Properties of VTA GABA Neurons

VTA GABA neurons are heterogeneous physiologically (Morales and Margolis, 2017). Electrophysiological experiments report a broad range of baseline firing rates of VTA GABA neurons, ranging from 2 to 20 Hz (Steffensen et al., 1998; Gallegos et al., 1999; Chieng et al., 2011; Cohen et al., 2012; Tan et al., 2012), which rise as high as 60 Hz in response to an aversive stimulus (Cohen et al., 2012; Tan et al., 2012). It is difficult to electrophysiologically distinguish VTA GABA neurons from neighboring VTA DA neurons (Margolis et al., 2012; Ungless and Grace, 2012). VTA GABA neurons generally fire faster than DA neurons, which typically fire between 1 and 10 Hz (Bunney et al., 1973; Grace and Bunney, 1984), and have shorter duration action potentials. While hyperpolarization-induced currents (I_h_) have traditionally been used to distinguish VTA DA neurons from VTA GABA neurons, it is a matter of debate whether these currents are expressed in VTA GABA neurons. In rats, most VTA GABA neurons express an I_h_ (Margolis et al., 2012). In a GAD2 green fluorescent protein (GFP) transgenic mouse line, however, VTA GABA neurons express very small, if any I_h_ (Chieng et al., 2011). In GAD1-GFP mice, VTA GABA neurons expressed I_h_ with amplitudes that were generally smaller than those of dopaminergic neurons, but still overlapped considerably (Merrill et al., 2015). It is possible that these discrepancies reflect differences between species and/or ages of animals or that GAD2-GFP or GAD1-GFP mice did not label 100% of GABA cells, and thus may be missing a subpopulation of GABA neurons that express an I_h_.

### Neurotransmitter Co-release From VTA GABA Neurons

A growing body of evidence indicates that midbrain neurons are rarely defined by a single neurotransmitter. Subpopulations of VTA neurons co-release both GABA and glutamate (Morales and Root, 2014; Yoo et al., 2016; Polter et al., 2018; Root et al., 2018). Many projections from the VTA to distal regions, including those to the LHb and the ventral pallidum, release both glutamate and GABA (Morales and Root, 2014; Yoo et al., 2016; Root et al., 2018). VTA and SNc dopaminergic neurons projecting to the striatum also co-release GABA at levels sufficient to suppress the output of striatal projection neurons (Kosaka et al., 1987; Tritsch and Sabatini, 2012). Interestingly, the production and maintenance of GABAergic release from dopaminergic neurons are not through canonical GAD1/2 pathways (Tritsch et al., 2014). Instead, the co-release of GABA from DA neurons is entirely dependent on alternative biochemical and uptake pathways. Midbrain DA neurons synthesize GABA through aldehyde dehydrogenase 1a1 (ALDH1a1; Kim et al., 2015). Likewise, dopaminergic neurons do not express VGAT, and vesicle loading of GABA is instead performed by the vesicular monoamine transporter (VMAT2; Tritsch et al., 2012). This co-release is maintained by plasma membrane uptake of GABA through mGAT1 and mGAT4, which are expressed in midbrain DA neurons (Tritsch et al., 2014). Because of these alternative pathways, VTA dopaminergic neurons do not express traditional markers of GABAergic identity such as GAD1/2 and VGAT and are thus not included in most studies of VTA GABA neurons, despite releasing GABA. Dopaminergic neurons that co-release GABA also co-release glutamate, providing a richly multiplexed system for regulation of striatal function. It remains unknown whether dopaminergic axons projecting to other regions such as the PFC or amygdala are also capable of co-releasing GABA, or if this is a unique function of mesostriatal neurons.

While it may seem counterintuitive for a neuron to co-release neurotransmitters with potentially opposing actions, this is a source of flexibility, providing several distinct mechanisms by which information can be transmitted through synapses. Glutamate and GABA typically accumulate into separate pools of synaptic vesicles, which means that the packaging, release, and recycling of each neurotransmitter can be differentially regulated (Root et al., 2018). Furthermore, when neurotransmitters are released from separate terminals, each type of terminal may be subject to neuromodulatory influences independent of the other. When glutamate and GABA are co-released at equal strength, it is then imperative on the postsynaptic cell to determine the cellular response. Dynamic regulation of membrane excitability or receptor availability will drastically alter how a cell responds to co-released neurotransmitters. Understanding how co-release of neurotransmitters impacts neuronal communication is one of the future key questions to understand the role this subset of cells plays in the regulation of the broader network.

## Behavioral Roles of VTA GABA Neurons

As potent regulators of VTA DA neurons and through their projections to distal brain regions, VTA GABA neurons are a critical hub for reward-related behavior. VTA GABA neurons are also highly sensitive to stressful and aversive stimuli. Given this regulation of reward and aversion, these neurons are poised to play a significant role in neuropsychiatric and substance use disorders.

### Modulation of Reward, Aversion, and Motivation

VTA GABA neurons are an important node in the brain’s reward processing networks. Optogenetic activation of VTA GABA neurons is sufficient to support real-time place aversion and to interrupt reward consumption measured in a cued- and free-access sucrose drinking paradigm (Tan et al., 2012; van Zessen et al., 2012). While both studies support an “anti-reward” effect of VTA GABA neuron stimulation, the behavioral paradigms used are quite different. In the first, the animals engaged in an active behavior to avoid stimulation of VTA GABA neurons during a real-time place preference (RTPP) task. In the second study, activation of VTA GABA neurons caused a passive pause in reward-consuming behavior. It could be that activating VTA GABA neurons induces an anhedonic state that is both aversive in nature and precludes reward consumption. Alternatively, these could represent distinct processes, one active and one passive, mediated by separate sets of projection or interneurons.

A large subset of VTA GABA neurons has been shown to be physically activated by reward-predictive cues (Pan et al., 2013). VTA GABA neurons have been proposed to encode the expectation of reward (Cohen et al., 2012). In an odor-cued conditioning task with rewarding or punishing outcomes, VTA GABA neurons displayed persistent activity during the delay period between a cue predicting the reward and the reward itself. In addition, when VTA GABA neurons were activated, VTA DA neurons responded to unexpected rewards as if they were expected (Eshel et al., 2015). Conversely, when VTA GABA neurons were inhibited, DA neurons responded to expected rewards as if they were unexpected (Eshel et al., 2015). Therefore, in this paradigm, VTA GABA neurons convey to DA neurons *how much* reward to expect. VTA GABA neurons inhibit DA neurons when reward is expected, causally contributing to prediction-error calculations. Additionally, bilateral stimulation of VTA GABA neurons reduces anticipatory licking to conditioned odors, consistent with these neurons playing a crucial role in reward-seeking (Eshel et al., 2015).

VTA GABA neurons also play a role in cue responsiveness, as chemogenetic activation of VTA GABA cell bodies disrupted responding to reward-signaling cues, although it did not affect motivation for the reward itself (Wakabayashi et al., 2019). Interestingly, locally activating VTA-originating GABAergic terminals in the NAc was not sufficient to disrupt cue responding, suggesting that the effects on cue responsiveness are mediated either by GABA interneurons within the VTA or by a those projecting to a different region of the brain.

VTA GABA neurons that project to the NAc have been shown to enhance associative learning through the silencing of cholinergic interneurons (Brown et al., 2012). Brown et al. (2012) show that VTA GABA neurons preferentially target cholinergic interneurons in the NAc (although others have shown robust innervation of striatal projection neurons as well, Ishikawa et al., 2013). Activation of VTA GABAergic projections in the NAc leads to a pause in the firing of cholinergic interneurons. This mimics the pause in cholinergic interneuron firing that often occurs during salient stimuli (Aosaki et al., 1994). Accordingly, when VTA GABAergic terminals were activated in the NAc during training to associate an auditory cue with a footshock, mice showed an enhanced ability to discriminate between conditioned and unconditioned stimuli (Brown et al., 2012).

Likewise, projections from the VTA to the LHb play a significant role in reward. These neurons, which co-release glutamate and GABA, have a net inhibitory effect on the activity of most habenular neurons (Root et al., 2014a,b; Yoo et al., 2016), although some neurons were excited (Root et al., 2014b). Photostimulation of this pathway in Vglut2-cre (a marker for glutamatergic neurons) or Vgat-Cre mice supports optical self-stimulation (Yoo et al., 2016). Intriguingly, mice prefer brief stimulation of this projection as opposed to sustained stimulation; accordingly, mice make more entries into the light-paired chamber in an RTPP assays, but spend less time in the chamber overall (Yoo et al., 2016), and develop conditioned aversion to a chamber paired with photostimulation (Root et al., 2014a). A similar mesohabenular projection has been described in TH-Cre mice (Stamatakis et al., 2013). These neurons express TH mRNA but do not release detectable DA in the habenula. Instead, neurons in this pathway release GABA (Stamatakis et al., 2013). Synaptic currents induced by photostimulation of this pathway were completely blocked by picrotoxin, suggesting that TH+ neurons exclusively release GABA. Like the Vglut2+ projection, photostimulation of this pathway supports optical self-stimulation, however unlike the Vglut2+ pathway, photostimulation of the TH+ pathway results in robust RTPP. This pro-reward effect is likely mediated through the silencing of the RMTg and increased activity of the VTA (Stamatakis and Stuber, 2012; Stamatakis et al., 2013). While these differences may arise from differences in experimental protocols or peculiarities of particular mouse lines, it is also possible that there are two parallel mesohabenular pathways with slightly divergent effects on the reward.

VTA GABA neurons projecting to the dorsal raphe also play a role in aversion and reward. This projection consists of two distinct, parallel pathways with opposing behavioral outputs. Activation of neurons in the rostral VTA that project to DRN serotonergic neurons is aversive in a RTPP assay, while activation of neurons in the caudal VTA that project to DRN GABA neurons is rewarding (Li et al., 2019). Taken together, these data suggest that projecting VTA GABA neurons have a significant and complex effect on reward and motivational processing and that discrete populations of these neurons support distinct behaviors.

Recent studies have also begun to dissect the functions of inputs onto VTA GABA neurons. For example, the photostimulation of the GABAergic component of the lateral hypothalamus-VTA pathway results in reward consummatory and social behaviors *via* inhibition of local VTA GABA interneurons and disinhibition of VTA DA neurons (Nieh et al., 2016). Likewise, photostimulation of the inhibitory projection from the BNST to the VTA results in RTPP and is sufficient to support self-administration. These effects are mimicked by photoinhibition of VTA GABA neurons (Jennings et al., 2013). Photostimulation of inhibitory projections from the MPOA to the VTA also stimulates RTPP, is sufficient for self-administration, and enhances female social attraction to a male (Tobiansky et al., 2013; McHenry et al., 2017). Because these studies each tested slightly different behaviors, it is unclear how much these behaviors depend on the pathway-specific engagement of VTA GABA neurons, for example, if photostimulation of MPOA GABAergic terminals would reduce anxiety in the elevated plus-maze or if photostimulation of BNST GABAergic terminals would promote social behavior. Nevertheless, it is clear that inhibition of VTA GABA neurons by inhibitory afferents broadly supports pro-reward behaviors.

As might be expected, activation of glutamatergic projections to VTA GABAergic neurons lead to aversive and defensive behavior. Vglut2 positive neurons projecting from the BNST to the VTA are excited by footshock and associated cues and activation of these terminals in the VTA promotes aversion in an RTPP assay and reduces center exploration of an open field (Jennings et al., 2013). Likewise, glutamatergic neurons projecting from the habenula to VTA and RMTg GABAergic neurons also drive aversion (Lammel et al., 2012; Stamatakis and Stuber, 2012). While this effect is primarily attributed to excitation of RMTg GABAergic neurons, it should be noted that the LHb also forms synapses on VTA GABA neurons (Omelchenko et al., 2009), and these could also play a role in driving aversion. A recent study demonstrated a role for glutamatergic control of VTA GABA neurons in defensive behaviors. Excitatory inputs from the superior colliculus to CeA-projecting VTA GABA neurons are activated by a looming stimulus mimicking an approaching aerial predator (Zhou et al., 2019). Inhibiting VTA GABA neurons decreases looming-evoked flight behavior, while photoactivation is sufficient to drive flight behavior. This suggests that VTA GABA neurons serve as a critical node in the integration of visual threat information and the expression of defensive-like behavior in response to an aversive visual stimulus.

### VTA GABA Neurons in Stress and Related Disease States

VTA GABA neurons are robustly activated by stressful or aversive stimuli. Acute exposure to foot shock, air-puff, or presentation of a looming stimulus that mimics an aerial predator leads to a drastic increase in the firing rate of VTA GABA neurons (Cohen et al., 2012; Tan et al., 2012; Zhou et al., 2019). In some studies, this increase is prolonged, lasting a full second beyond footshock onset (Tan et al., 2012). Likewise, inputs to VTA GABA neurons are also highly responsive to stress. For example, the firing rate of VTA-projecting GABA neurons in the BNST, which preferentially target GABA neurons, is sharply decreased during footshock or a footshock-associated cue (Jennings et al., 2013). In contrast, BNST glutamatergic neurons that target VTA GABA neurons have an increase in firing rate during footshock or a footshock-associated cue. Taken together, this suggests that acute stress causes a concomitant decrease in inhibition and increase in excitation from the BNST onto VTA GABA neurons, likely resulting in the increased firing of VTA GABA neurons.

While acute stress has an immediate effect of increasing firing of VTA GABA neurons, the lasting effects of acute stress are more complicated as the plasticity of GABAergic synapses onto DA neurons is affected over a longer timescale. Nitric-oxide dependent long-term potentiation of GABAergic synapses (LTP_GABA_) is induced by NMDA receptor activation (Nugent et al., 2007), potentially serving as a brake on DA neuron firing in the face of strong excitatory activity. This form of LTP is at least partially mediated by local synapses arising from VTA GABA neurons (Simmons et al., 2017; Polter et al., 2018). Following acute swim stress, LTP_GABA_ is blocked for up to a week through activation of kappa opioid receptors (Niehaus et al., 2010; Graziane et al., 2013; Polter et al., 2014, 2017). Therefore, although the immediate effect of acute stress is an increase in the activity of VTA GABAergic neurons and a suppression of dopaminergic firing rate, over the following days GABAergic plasticity is lost, removing an important brake on dopaminergic firing. In contrast, others have observed enhanced VTA GABAergic inhibition of DA neurons that lasts at least a day after single restraint stress (Ostroumov et al., 2016). This occurs through a shift in the chloride reversal potential that leads to GABAergic currents onto VTA GABA neurons becoming excitatory rather than inhibitory. While these results are somewhat contradictory, these studies differ in the type and duration of stress. Prior studies have shown vastly different effects of different stressors on VTA DA neurons (Chaudhury et al., 2013; Tye et al., 2013). It should come as no surprise that VTA GABA neurons have a similarly complex relationship with stress. Human studies and animal models have long indicated an interaction between stress and neuropsychiatric disorders such as depression, anxiety, and PTSD. Depressed human subjects show disruptions in processing of reward and punishment, and have abnormalities in the function of brain areas innervated by monoamines (Eshel and Roiser, 2010). Through their regulation of VTA DA neurons and modulation of reward and punishment, VTA GABA neurons are prime candidates for contributing to stress-linked disorders such as depression, as they represent a key link between stressful experiences and hedonic behavior.

Stress is also a contributing factor to substance use disorders. Stress promotes both opioid and psychostimulant abuse in humans (Sinha, 2008) and acute or chronic stress has been shown to facilitate the acquisition of drug self-administration, enhance behavioral sensitization and to cause the reinstatement of drug-seeking (Piazza et al., 1990; Piazza and Le Moal, 1998; Mantsch et al., 2016). As with acute stress, acute exposure to drugs of abuse can change the activity of VTA GABA neurons. For example, opioids hyperpolarize VTA GABA neurons, disinhibiting VTA DA neurons (Johnson and North, 1992), although recent studies have suggested that RMTg GABA neurons, rather than VTA GABA neurons are the major opioid-sensitive inhibitory input onto VTA DA neurons (Matsui and Williams, 2011; Matsui et al., 2014). Others have suggested that opioids may alter chloride reversal potentials in VTA GABA neurons, leading to excitatory GABAergic transmission onto VTA GABA neurons (Ting-A-Kee et al., 2013). Δ9-tetrahydrocannabinol (THC) depresses VTA GABA neuron activity, leading to a disinhibition of VTA DA cells (Friend et al., 2017). Acute ethanol increased the firing rate of VTA GABA neurons (Steffensen et al., 2009) and increases the frequency of EPSCs but inhibited mEPSC frequency and amplitude onto VTA GABA neurons in a rat model (Williams et al., 2018).

Drugs of abuse also acutely affect synaptic plasticity of VTA GABA neurons, as a single exposure to morphine, cocaine, ethanol or nicotine is able to block GABAergic LTP on DA neurons (Nugent et al., 2007; Nugent and Kauer, 2008; Guan and Ye, 2010; Niehaus et al., 2010). Only a few studies have looked at repeated exposure to drugs of abuse and VTA GABA neurons. Five days of cocaine injections occluded cAMP-induced LTP of NAc-originating inhibitory synapses onto VTA GABA neurons (Bocklisch et al., 2013). Optogenetically inducing this LTP *in vivo* leads to a decreased firing rate for VTA GABA neurons, disinhibition of VTA DA neurons, and a days-long enhancement of conditioned place preference to cocaine. Chronic, but not acute, exposure to THC occludes long term depression of excitatory synapses onto VTA GABA neurons (Friend et al., 2017). While these articles focus on the effects of different drugs of abuse, both report alterations in neurotransmission that would result in disinhibition of DA neurons. Similarly, silencing VTA GABA neurons by virally expressing tetanus toxin leads to an enhanced sensitization to cocaine (Gore et al., 2017). In contrast, chronic ethanol exposure increases excitatory tone onto VTA GABA neurons, suggesting increased excitability of these cells (Williams et al., 2018). This difference may represent differences between the mechanisms of distinct drugs of abuse or it may be due to the selection of distinct populations of VTA GABA neurons. These data suggest that VTA GABA neurons are an important substrate for drugs of abuse, and targeting them may be a fruitful strategy to treat substance use disorders.

## Discussion and Future Directions

### VTA GABA Neurons as a Gate of Dopaminergic Activity

A number of studies in recent years have suggested that VTA GABA neurons function as a gate, tonically suppressing VTA DA neurons and releasing this inhibition when they themselves are inhibited by projections from distal brain regions. This is a repeated pattern, as inhibitory projections from the BNST, LH, NAc and MPOA form GABA_A_ synapses on VTA GABA neurons (Jennings et al., 2013; Nieh et al., 2016; McHenry et al., 2017). Activation of these projections, or inhibition of VTA GABA neurons, results in general pro-reward behavior, reduced anxiety, an increase in social interaction, reward consumption, and an increase in exploration.

Although there is strong behavioral evidence in favor of this gating model, the physiological evidence is a little less straightforward. While there are several studies that show that VTA GABA neurons inhibit firing of VTA DA neurons or release of DA in target areas, the magnitude of this inhibition varies considerably. In one such study, *in vivo* optogenetic stimulation of VTA GABA neurons resulted in a near-total suppression of VTA DA neuron firing (Tan et al., 2012). In another, optogenetic stimulation of VTA GABA neurons in acute slices decreases firing by approximately 50% in response to an injected current ramp, and partially decreased evoked DA release in the NAc at low, but not high, stimulation frequencies (van Zessen et al., 2012). Another report shows that optogenetic activation of VTA GABA neurons leads to a relatively modest decrease in baseline and reward-evoked firing of VTA DA neurons (Eshel et al., 2015). Finally, a recent report demonstrated a small effect of optogenetic activation of VTA GABA neurons on firing rates in slices (~25%, Simmons et al., 2017). These differences may reflect differences in experimental preparation, however, it is clear that while VTA GABA neurons are capable of completely silencing VTA DA neurons, under other conditions they have a relatively modest effect on firing of DA neurons.

To truly model gating of VTA DA neurons by VTA GABA neurons, it is, of course, essential to demonstrate that *inhibition* of VTA GABA neurons leads to an increase in the firing rate of DA neurons. To the best of our knowledge, only one such study has been performed. Eshel and colleagues found that optogenetically inhibiting VTA GABA neurons led to an increase in firing in a minority of DA neurons (Eshel et al., 2015). However, this experiment is somewhat confounded by the fact that optogenetic inhibition of VTA GABA neurons led to only a modest reduction in the firing rate of VTA GABA neurons, with significant activity persisting through the stimulation. Understanding how VTA GABA neurons gate activity of VTA DA neurons and thus reward will require improved optogenetic inhibitors or use of chemogenetics or of selective lesion studies.

Intriguingly, inducing LTP of VTA GABAergic synapses onto DA neurons greatly increased the efficacy of inhibition of DA neurons by optogenetic activation of VTA GABA neurons (Simmons et al., 2017). This suggests that the ability of VTA GABA neurons to suppress the firing of VTA DA neurons may be state-dependent. This is particularly intriguing given that this form of LTP_GABA_ is blocked a single administration of drugs of abuse such as morphine, cocaine, and ethanol (Nugent et al., 2007; Guan and Ye, 2010; Niehaus et al., 2010) or by a single exposure to an acute forced swim stressor (Niehaus et al., 2010). Our prior work shows that this suppression of LTP by stress lasts for nearly a week after a single stressor (Polter et al., 2017). Therefore it is possible that a single adverse event could lead to a days-long change in the ability of VTA GABA neurons to suppress the firing of neighboring neurons.

### Unexplored Facets of Regulation of VTA GABA Neurons

Most existing studies of VTA GABA neurons have focused on their regulation by acute stimuli or their role in short-term behavioral paradigms. However, given their critical role in encoding aversion and gating reward, it is important to better understand the dynamic regulation of these cells by chronic stressors and long-term exposure to drugs of abuse. It is also vital to look at how VTA GABA neurons change between acute and long-term stress or drug exposure to better understand how and why this transition leads to both adaptive and maladaptive behaviors.

As we consider the role that VTA GABA neurons play in stress-related pathologies, there are several variables to consider. The first is that of sex: stress-linked disorders are highly sexually dimorphic (Piccinelli and Gomez Homen, 1997; Bigos et al., 2009), and it will be important to understand the regulation of VTA GABA neurons in both sexes. Multiple lines of evidence suggest that these neurons may be regulated in a sexually dimorphic fashion. For example, VTA DA neurons fire faster in females in estrus than in females in diestrus or males (Zhang et al., 2008; Calipari et al., 2017), it is possible that VTA GABA neurons are likewise regulated by the estrus cycle. Furthermore, many inputs that VTA GABA neurons receive such as those from the BNST and the mPOA are themselves sexually dimorphic, suggesting that VTA GABA neurons of males and females may receive inputs that are differentially regulated (Jennings et al., 2013; McHenry et al., 2017). In addition to sex, it is also important to consider development and age as a factor in the regulation of VTA GABA neurons. DA neurons undergo dynamic changes in activity across development, and it is important to understand the role shifts in inhibitory tone could play in this. For example, DA neurons fire faster in adolescence, an effect that is concomitant with decreased spontaneous inhibitory transmission (McCutcheon et al., 2012). Studies in the visual system have shown that GABAergic inhibition is an important driver of circuit development (Fagiolini and Hensch, 2000), it is possible that this is true for the VTA as well. Understanding developmental changes in VTA inhibitory circuitry is particularly important because we know that there are developmental sensitive periods during which exposure to stress or drugs of abuse can lead to lasting changes in the function of the VTA (Peña et al., 2017; Frau et al., 2019).

Another significant factor in the regulation of VTA GABA neurons is that of sleep, arousal, and circadian rhythms. VTA DA neurons play a significant role in sleep and wakefulness, exhibiting higher activity during wakefulness (Eban-Rothschild et al., 2016; Fifel et al., 2018). A growing body of evidence points toward the role of VTA GABA neurons in these processes (Takata et al., 2018; Yu et al., 2019). Indeed, when activated optogenetically, those neurons produce a profound sedative state. Lesion of these neurons causes a permanent sleep loss that persists for months (Yu et al., 2019). VTA GABA neurons limit wakefulness both *via* projections to arousal-promoting orexin neurons in the LH and by inhibiting glutamatergic and DA cells locally in the VTA. VTA DA neurons are also modulated by circadian genes, such as the transcription factor CLOCK (McClung et al., 2005), however it remains unknown whether activity of VTA GABAergic neurons is regulated by these genes or exhibit shifts in activity across the circadian cycle. Bipolar disorder, depression or substance use disorder have been associated with disruption in sleep and circadian rhythmicity (McClung et al., 2005; Sidor et al., 2015; Satyanarayanan et al., 2018; Caumo et al., 2019). Given the bidirectional relationship between circadian rhythms and sleep and the VTA, dysregulation of VTA function is an attractive mechanism for circadian and arousal deficits in mood and substance use disorders. The ability of VTA GABA neurons to modulate sleep and arousal should also give pause when interpreting results of behavioral tests after manipulation of VTA GABA neurons, as it is important to consider whether changes in reward pursuit or anxiety-like behavior might not instead be caused by changes in arousal state.

### Underappreciated Heterogeneity of VTA GABA Neurons

A critical piece moving forward will be to understand diversity among VTA GABA neurons, as well as developing a deeper understanding of the role of inhibition in the VTA. There are several lines of circumstantial evidence that suggest that there are subdivisions among VTA GABA neurons. The first is that there seem to be distinctions between projection neurons and local inhibitory interneurons. Second, there are a number of physiological measures of GABA neurons that have a high degree of variability; for example, the literature contains a wide range of baseline firing rates of GABA neurons (Steffensen et al., 1998; Gallegos et al., 1999; Chieng et al., 2011; Cohen et al., 2012; Tan et al., 2012), and the response to opioid agonists at VTA GABA neuron terminals varies considerably more than the response at terminals arising in the RMTg and NAc (Matsui et al., 2014). Efforts to measure the expression of peptide and calcium-binding protein markers have also seen high variability within VTA GABA neurons (Olson and Nestler, 2007; Merrill et al., 2015). Finally, recent work has laid out the elegant circuit logic of the DA neurons of the VTA. Segregated populations of DA neurons project from the VTA to the medial accumbens, lateral accumbens, basolateral amygdala, and medial PFC (Lammel et al., 2014). These projections encode opposing behavioral states, receive discrete inputs, and are activated by distinct stimuli (Lammel et al., 2012, 2014). It would, therefore, be surprising, although not impossible, for these neurons to receive identical local inhibitory input.

It is also imperative to learn more about the properties of projecting VTA GABA neurons. While the anatomy and behavioral functions of individual projections have been reported, there is still much to be studied. Functional studies to illuminate the physiology and modulation by the experience of these neurons will be important. It will also be critical to understand how these projections interact, and whether there are collateralized projections that target multiple brain regions. Likewise, projection neurons may have local collaterals, allowing coordination of VTA activity with activity in distal target regions. Furthermore, it is unclear how homogeneous each projection is. For example, the projection from the VTA to the dorsal raphe has two functional branches that target different cells and have opposing effects on behavior. Other projections may have similar heterogeneity.

To gain a full understanding of the functions of subgroups of VTA GABA neurons, it will be necessary to gain genetic access to these cells. For projection neurons, this is possible to do using retrograde viruses. However, for local neurons, and for projections containing heterogeneous cells, it will require isolating cell-type-specific genetic markers. Attempts to sub-classify VTA GABA neurons using markers from the forebrain have thus far been unsuccessful, as might be expected given that VTA GABA neurons and forebrain GABA neurons arise from distinct developmental lineages. Recent work utilizing modern genetic tools to identify potential novel markers of VTA GABAergic subclasses is an important start towards gaining genetic access (Paul et al., 2018, 2019; Table 1).

As the subpopulations of VTA GABA neurons are identified, it will be important to thoughtfully consider the role they play within the VTA microcircuit and in regulation of distal circuits. As is seen in forebrain structures, the roles that inhibitory neurons can play within a circuit are rarely as straightforward as simply stopping a postsynaptic neuron from firing. In cortical and hippocampal regions, we know that synapses arising from interneurons can modulate integration of excitatory currents in dendrites, tune temporal fidelity of signals through feed-forward inhibition, inhibit other interneurons to disinhibit pyramidal cells, or release GABA extra-synaptically to provide an ambient level of tonic inhibition (Wamsley and Fishell, 2017). It will be critical going forward to integrate this information into studies of VTA GABA neurons to develop a complete model of how inhibition in the VTA guides signal processing by VTA DA neurons. As we better understand the specific roles subtypes of GABA neurons play within the VTA and throughout the brain, we will gain a better understanding of specific mechanisms of processing reward and aversion. Unraveling the complexity of the VTA microcircuit will be a challenging but important step to understand how these neurons regulate reward, motivation, cognition, aversion, and affect in both health and disease.

## Author Contributions

CB and BT drafted the manuscript. AP edited the manuscript.

## Conflict of Interest

The authors declare that the research was conducted in the absence of any commercial or financial relationships that could be construed as a potential conflict of interest.
